# Acute Effects of Hamstring Stretching on Sagittal Spinal Curvatures and Pelvic Tilt

**DOI:** 10.2478/v10078-012-0007-7

**Published:** 2012-04-03

**Authors:** Pedro A. López-Miñarro, José M. Muyor, Felipe Belmonte, Fernando Alacid

**Affiliations:** 1 Department of Physical Education. University of Murcia.; 2 Department of Physical Education. University of Almería.; 3 Department of Physical Activity and Sports. University of Murcia.

**Keywords:** flexibility, posture, thoracic, lumbar, spine

## Abstract

The aim of this study was to determine acute effects of hamstring stretching in thoracic and lumbar spinal curvatures and pelvic tilt. Fifty-five adults (29.24 ± 7.41 years) were recruited for this study. Subjects performed a hamstring stretching protocol consisting of four exercises. The session consisted of 3 sets of each exercise and subjects held the position for 20 seconds with a 30-second rest period between sets and exercises. Thoracic and lumbar spinal angles and pelvic tilt were measured with a SpinalMouse in relaxed standing, sit-and-reach test and Macrae & Wright position. Hamstring extensibility was determined by active straight leg raise test and sit-and-reach score. All measures were performed before and immediately after the hamstring stretching protocol. Active straight leg raise angle and sitand-reach score significantly improved immediately after the stretching protocol (p<0.001). Greater anterior pelvic tilt (p<0.001) and lumbar flexion (p<0.05) and a smaller thoracic kyphosis in the sit-and-reach (p<0.001) were found after the stretching protocol. However, stretching produced no significant change on spinal curvatures or pelvic tilt in standing and maximal trunk flexion with knees flexed. In conclusion, static stretching of the hamstring is associated to an immediate change in the sagittal spinal curvatures and pelvic position when performing trunk flexion with knees extended, so that allowing for greater lumbar flexion and anterior pelvic tilt and lower thoracic kyphosis. Hamstring stretching is recommended prior to sport activities involving trunk flexion with the knees straight.

## Introduction

The effects of stretching exercises on hamstring extensibility are a topic of continued interest to researchers. Stretching can provide a range of health-related motion benefits. Clinicians have generally considered flexibility training to be an integral component in the prevention and rehabilitation of injuries, as well as a method of improving performance in daily activities and sports. Hamstring flexibility is an important variable because reduced extensibility has been proposed as a predisposing factor for injuries (Hartig and Herderson, 1999), non-specific low back pain (Jones et al., 2005), and changes in lumbopelvic rhythm (Esola et al., 1996).

Several studies have analyzed the acute effects of hamstring stretching on extensibility (Nelson, 2006; Youdas et al., 2010; O’hora et al., 2011; Puentedura et al., 2011). Hamstring stretching has been related to an acute improvement in hip flexion with knee extended (straight leg raise test) or active and passive knee extension with the hip flexed to 90º (Halbertsma et al., 1996; Spernoga et al., 2001).

Hamstring muscles attach proximally to the ischial tuberosity, except for the short head of biceps femoris. Because the hamstring muscles originate at the ischial tuberosity of the pelvis, the tension in the hamstring muscles has an influence on pelvic posture (Congdon et al., 2005). The pelvis is considered to be the base for the spine, and its anteroposterior orientation affects the sagittal curves of the spine (Delisle et al., 1997). For this reason, a change in hamstring extensibility should have some influence in pelvic and spinal postures when the hamstring muscles are subjected to moderate or high tension.

Previous studies have analyzed the influence of hamstring extensibility on spinal and pelvic postures between healthy subjects and athletes of differing flexibility (Gajdosik et al., 1994; Carregaro and Coury, 2009; López-Miñarro et al., 2011; Muyor et al., 2011). Other studies have found that hamstring extensibility influences the thoracic and pelvic postures in athletes when the sit-and-reach test was performed (López-Miñarro et al., 2009; López-Miñarro and Alacid, 2010; López-Miñarro et al., 2010). All these studies were cross-sectional and no acute intervention was carried out. Borman et al. (2011) determined the effect of stretch position on hamstring muscle extensibility, lumbar flexion range of motion and lumbar curvature after 4 weeks. They found no change for lumbar flexion range of motion or in lumbar curvature even though hamstring muscle extensibility increased.

Furthermore, to date, no studies have specifically examined the acute effects of hamstring stretching in sagittal spinal curvatures and pelvic tilt. Therefore, the purpose of this study was to determine the acute effects of hamstring stretching in thoracic and lumbar spinal curvatures and pelvic tilt in standing and trunk flexion postures.

## Material and Methods

### Participants

Fifty-five employees of the police force service in an urban area (29.24 ± 7.41 years, body height 178.64 ± 5.14 cm, body mass 80.72 ± 9.50 kg) were recruited. Subjects practiced recreational physical exercise but were not participating in any structured flexibility training at the time of the study. All the subjects were informed of the nature and the aim of this study before they signed an informed consent form. This study was approved by the Ethics and Research Committee of the University of Murcia.

All subjects were asked to complete a health-screening questionnaire about their physical activity pattern, previous hamstring injuries and stretching and low back pain history. Subjects were excluded if (1) they had had any hamstring injury (hamstring muscle strain, hamstring spasm, or tendinopathies) within the last 6 months or (2) they had a history of low back pain in the last 2 months.

### Procedures

Sagittal spinal curvatures and pelvic tilt were measured in the relaxed standing, sit-and-reach test and Macrae & Wright test. Hamstring muscle extensibility was determined in both legs by active straight leg raise test and the sit-and-reach score. Three trials for each measure were administered, and the average value of measures was entered for data analysis. The measurements were made in a randomized order. The subjects were allowed to rest briefly standing up for 5 minutes between measures in the pre-test. At post-test the measures were immediately made without rest to avoid a decrease of extensibility. All measurements were made during the same testing session and were administered under the same environmental conditions. Participants were instructed not to undertake a weight-training session or strenuous exercise the day before testing to ensure consistent test conditions. No warm-up was performed by the subjects prior to the test measurements. The subjects wore underclothing and no shoes. All measurements were taken around midday on each of the testing days (between 11:00 and 13:00 h) to control diurnal variations in spinal curvatures and hamstring extensibility.

Spinal curvatures and pelvic tilt were measured using a SpinalMouse system (Idiag, Fehraltdorf, Switzerland). The SpinalMouse is an electronic computer-aided measuring device which measures sagittal spinal range of motion and intersegmental angles in a non-invasive way and is a so-called surface-based technique. The device is connected via an analog-digital converter to a standard PC. For global spinal angles, the Spinal Mouse is a valid and reliable device (Guermazi et al., 2006; Mannion et al., 2004).

Prior to taking measurements, the main researcher determined the spinous process of C7 (starting point) and the top of the anal crease (end point) by palpation and marked the skin surface with a pencil. The Spinal Mouse was guided along the midline of the spine (or slightly paravertebrally, particularly in thin individuals with prominent spinous process) starting at the spinous process of C7 and finishing at the top of the anal crease (approximately S3). For each testing position, the position of the thoracic (T1–2 to T11–12) and lumbar (T12-L1 to the sacrum) spine and the position of the sacrum and the hips (difference between the sacral angle and the vertical) were recorded. In the lumbar curve negative values corresponded to lumbar lordosis (posterior concavity). With respect to the pelvic tilt, a value of 0º represented the vertical position. Thus, a greater angle reflected an anterior pelvic tilt, and a lower angle (negative values) reflected a posterior pelvic tilt.

### Measures

#### Hamstring muscle extensibility

Hamstring extensibility was determined by performing an active straight leg raise (ASLR) on each limb in a counterbalanced order. While the participant was in the supine position, the axis of a Universal goniometer was aligned with the axis of the hip joint. The tester placed the stationary arm in line with the trunk and positioned the moveable arm in line with the femur. The participant’s leg was lifted passively by the tester into hip flexion until the subjects reported pain in their hamstring. The knee remained straight during the leg raise. The ankle of the tested leg was positioned in maximal plantar flexion to avoid adverse neural tension. Moreover, the pelvis was fixed to avoid the posterior pelvic tilt and an auxiliary tester kept the contralateral leg straight to avoid external rotation. A Velcro strap was positioned over the anterior superior iliac spine to limit posterior pelvic tilt. The criterion score of hamstring extensibility was the maximum angle (degree) read from the goniometer at the point of maximum hip flexion. Angles were recorded to the nearest degree for each leg.

#### Sit-and-reach test

**Figure 1:**
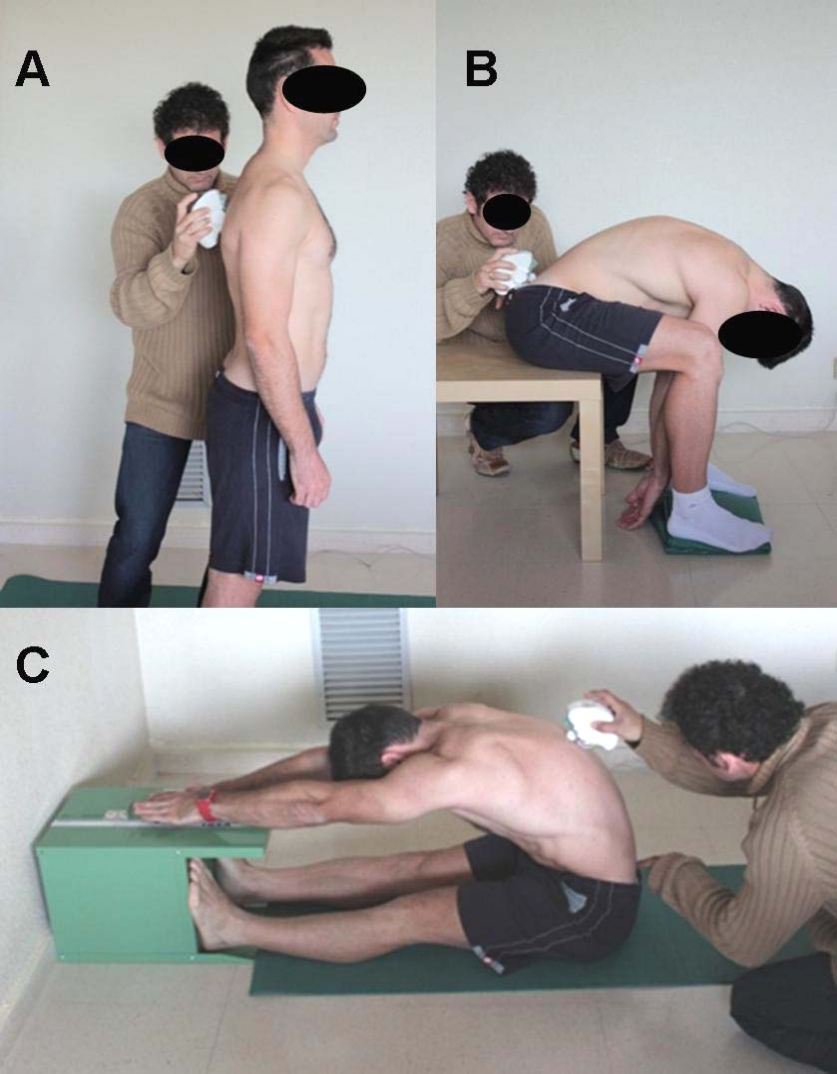
Spinal measurement in standing (A), Macrae & Wright test (B) and sit-and-reach test (C)

Spinal angles, pelvic tilt and score were measured in the sit-and-reach test when the subjects reached the maximal trunk flexion with knees extended (Figure 1). The subjects were required to sit with knees straight and legs together so that the soles of the feet were flat against the end of a constructed box (ACUFLEX I Flexibility tester, height = 32 cm). With palms down, placing one hand on top of the other, the subjects slowly reached forward as far as possible sliding the hands along the box with the knees as straight as possible and held the position for approximately five seconds while the spinal curvatures, pelvic tilt and score were measured. The distance from the toes (zero point) was measured in centimeters. The score was the greatest distance contacted by the fingertips and was registered with an accuracy of up to 0.5 cm. Positive values were awarded if subjects could reach beyond their toes, and negative values were awarded if subjects could not reach beyond their toes.

#### Macrae & Wright test

The subjects with knees flexed (90º) in a sitting position were asked to bend forward maximally (Macrae & Wright, 1969) (Figure 1). Spinal angles and pelvic tilt were measured when the subjects reached maximal trunk flexion.

#### Standing

The subject assumed a relaxed position, with the head looking forward, arms hanging by the sides, knees normally extended and the feet shoulder-width apart (Figure 1).

#### Stretching protocol

**Figure 2:**
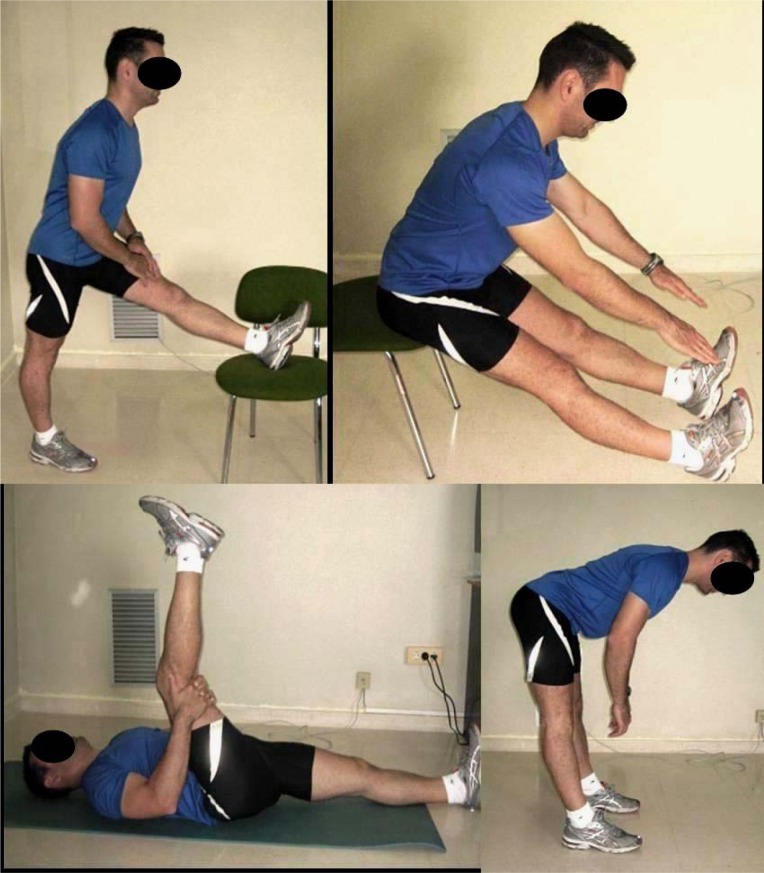
Hamstring stretching exercises

The stretching exercises were performed according to the static method in the following order: 1) unilateral standing hamstring stretch; 2) bilateral stretching while sitting in a stool; 3) unilateral stretching while lying supine; and 4) bilateral standing hamstring stretch (Figure 2). Verbal feedback was given throughout the stretching protocol to ensure that proper technique was maintained. In all stretching exercises the subjects flexed their hip or trunk, maintaining their spine as aligned as possible until a mild stretch sensation was felt in the posterior thigh. The session consisted of 3 sets of each exercise and the position was held for 20 seconds with a 30-second rest between sets and exercises. The training session lasted approximately 8 minutes.

### Analysis

The results of a 2-tailed t test for dependent samples with the alpha set at 0.05 revealed that a sample size of 51 was needed to achieve 90% power. The hypotheses of normality and homogeneity of variance were analyzed using the Kolmogorov-Smirnov test and Levene’s test, respectively. Statistical tests revealed no violations of the assumptions of normality and homogeneity. Parametric analysis was performed. Means and standard deviations were calculated for all variables. A dependent t-test was conducted to determine whether a significant difference existed between pre- and post-stretching values for all dependent variables. We also calculated the minimal detectable change (MDC), which was used to determine the smallest change necessary for declaration of differences between measurements. The minimal detectable change at the 95% confidence level (MDC95%) was calculated using the formula MDC95% = SEM * 1.96 * √2. The standard error of measurement (SEM) was calculated using the formula SEM = SD * √1 − *R*. The SD means the standard deviation of all scores of the 3 assessments, and R was the intraclass correlation coefficient (ICC 3,1) according to the formula described by Shrout and Fleiss (1979). The alpha level was set at 0.05. Analyses were performed using the SPSS 15.0 statistical software package.

## Results

The mean values of the active straight leg raise test and sit-and-reach score in the pre- and post-stretching are shown in Table 1. Significant improvements were found in both measures.

The thoracic and lumbar angles and pelvic tilt in standing, sit-and-reach test and Macrae & Wright test are presented in Table 2. The sit-and-reach test showed a significant change in spinal angles and pelvic tilt. An increase in the anterior pelvic tilt and lumbar flexion was found (*p*<0.001). By contrast, there was a significant decrease in thoracic kyphosis (Table 2). No significant differences were found between preand post-stretching measures in the standing and Macrae & Wright test.

## Discussion

The main purpose of this study was to determine the acute effects of hamstring stretching in thoracic and lumbar spinal curvatures and pelvic tilt. The purpose of stretching is most usually to increase the range of motion and reduce tension and stiffness of the muscle-tendon unit. However, the acute influence of a stretching protocol in sagittal spinal curvatures and pelvic tilt has not previously been analyzed. The findings of this study demonstrate significant increases in hip flexion range of motion in the active straight leg raise test immediately after the stretching protocol. This change was associated to a significant improvement in anterior pelvic tilt and lumbar flexion as well as reduced thoracic kyphosis in the sit-and-reach test. Kippers and Parker (1987) indicated that when the trunk is inclined well past the vertical plane as a result of hip movement, maximum reach may be achieved by less than maximal vertebral flexion.

These findings are important in sport activities that involve trunk flexion movements with knees extended or slightly bent because a reduction in thoracic kyphosis and improved anterior pelvic tilt after stretching can be achieved. Sagittal curvatures are geometric parameters which influence mechanical properties during compressive loading (Keller et al., 2005). Greater spinal angles produce larger shear forces, larger contributions from passive components (McGill, 2002), and greater intradiscal pressures on the thoracic and lumbar tissues (Wilke et al., 2001; Polga et al., 2004) predisposing the subjects to spinal disorders (Smith et al., 2008).

Some studies found a trend in subjects with greater hamstring extensibility towards greater anterior pelvic tilt and lower thoracic flexion angle when maximal trunk flexion with knees extended was performed (Gajdosik et al., 1994; López-Miñarro and Alacid, 2010; López-Miñarro et al., 2010). However, the design of these studies was cross-sectional and did not include an intervention. Borman et al. (2011) found no change in lumbar flexion range of motion or in lumbar curvature even though hamstring muscle extensibility increased after 4 weeks. Li et al. (1996) found that straight leg raising and hip motion during late and total forward bending were increased after stretching program lasting 3 weeks. Furthermore, no changes were found in lumbar motion during trunk forward bending.

In the current study improved hamstring extensibility had no effect on spinal curvatures and pelvic tilt in either the standing position or maximal trunk flexion in sitting with knees flexed at 90 degrees (Macrae & Wright position). The flexed position of the both knees while maximal trunk flexion is performed reduces the tension on hamstring muscles and limits its influence on the pelvis and sagittal spinal curvatures. In accordance with previous cross-sectional studies (Gajdosik et al., 1994; López-Miñarro and Alacid, 2010; Li et al., 1996), the thoracic and lumbar angles and pelvic tilt in standing are not influenced by hamstring extensibility, because the hamstring muscles are slightly extended with little passive tension.

Several studies have found a significant improvement in active knee extension test in young adults (between 5–12º) immediately after a hamstring stretching protocol or single stretching (DePino et al., 2000; Spernoga et al., 2001; Nelson, 2006; Youdas et al., 2010; O’hora et al., 2011; Puentedura et al., 2011). In the current study an improvement around 9º in the active straight leg raise test was found. Furthermore, an improvement in the sit-and-reach score was detected (mean difference pre-post score: +5.45 cm). The greater score after stretching was related to improved anterior pelvic tilt and greater lumbar flexion. Prior to hamstring stretching exercises the subjects showed greater posterior pelvic tilt in the sit-and-reach. Increased hamstring extensibility after stretching increased anterior pelvic tilting when maximal trunk flexion with knees extended was performed. As the participant bends forward, the pelvis rotates further forward until the passive tension in the hamstrings limits pelvic rotation (McGill, 2002; Peharec et al., 2007). Some research has suggested that the increases in range of motion observed following an acute stretching intervention are possible because the subject has adapted to this discomfort and therefore is more tolerant of the stretch discomfort in the new range (Halbertsma et al., 1996; Magnusson et al., 1996). Spernoga et al. (2001) suggests that a single session of stretching does not deform tissues enough to produce a permanent adaptive change.

With regard to methodological considerations, we selected static stretching because it is a common technique used by strength and conditioning specialists and athletes to increase muscle length. This type of stretching has been shown to be effective in increasing hamstring length (Davis et al., 2005). However, some studies have compared the effects of other stretching techniques on the length of the hamstring muscle group during a short-term training program and detected different effects according to the technique used (Davis et al., 2005; Youdas et al., 2010; O’hora et al., 2011). Further studies should examine the influence of using the stretching technique used on spinal curvatures and pelvic posture.

The main limitation of the study was not to conduct an analysis of the duration of the changes in extensibility and spinal curvatures. Previous studies have found that the effect of hamstring stretching in active knee extension test is limited and lasts around 3–6 minutes after cessation of the stretching protocol (DePino et al., 2000; Spernoga et al., 2001). Future studies should address the duration of improvement in spinal and pelvic postures after stretching cessation.

The present study has another limitation that need to be taken into account when considering the study and its contributions. Pre-exercise routines are common practice for the majority of individuals participating in physical exercise. Stretching exercises are either performed alone or with other exercises as part of the warm-up. An aerobic warm-up is usually used as it has been shown to improve hamstring extensibility in combination or not with static stretching (Murphy et al., 2010; Perrier et al., 2011). In contrast, other studies have found that a warm-up exercise prior to stretching does not appear to significantly increase the effectiveness of static hamstring stretching (Weijer et al., 2003; O’Sullivan et al., 2009). The current study only examined the isolated effects of static stretching on hamstring muscle extensibility and spinal posture because a lot of subjects only incorporate stretching exercise previously to their exercise sessions. However, future studies should analyze the influence of aerobic warm-up alone or combined with stretching in hamstring muscle extensibility, spinal curvatures and pelvic inclination to determine the most effective protocol.

In conclusion, static stretching of the hamstring muscles is associated with immediate changes in the sagittal spinal curvatures and pelvic position, allowing for greater lumbar flexion and anterior pelvic tilt as well as lower thoracic kyphosis when performing maximum trunk flexion with knees extended. Hamstring stretching is recommended prior to sport activities involving trunk flexion with the knees straight.

## Figures and Tables

**Table 1:** Mean (± standard deviation) of active straight leg raise test and sitand-reach score prior and after hamstring stretching

Test	Pre-stretching	Post-stretching	Mean difference	t	p-value	MDC
ASLR right	68.61 ± 11.76º	77.51 ± 10.58º	8.90 ± 4.94º	−12.846	< 0.001	4.68º
ASLR left	68.82 ± 12.20º	77.53 ± 10.84º	8.71 ± 5.99º	−10.372	< 0.001	4.53º
SR (cm)	−2.84 ± 10.53	2.61 ± 10.43	5.45 ± 2.41	−16.091	< 0.001	4.05

ASLR: active straight leg raise test; MDC: minimal detectable change; SR: sit-and-reach test

**Table 2:** Mean (± standard deviations) values for the thoracic spine, lumbar spine and pelvic tilt before and immediately after stretching protocol

Variable	Pre-stretching	Post-stretching	Mean difference	t	p-value	MDC
**Standing**						
Thoracic spine	43.92 ± 10.49º	42.45 ± 11.04º	−1.47 ± 8.74º	1.201	NS	4.05º
Lumbar spine	−24.71 ± 6.75º	−24.69 ± 9.28º	−0.02 ± 6.87º	−0.020	NS	2.59º
Pelvic tilt	11.29 ± 4.88º	11.76 ± 6.43º	0.47 ± 3.13º	−1.073	NS	1.73º
**Sit-and-reach test**						
Thoracic spine	70.00 ± 11.39º	63.55 ± 11.45º	−6.45 ± 7.33º	6.278	< 0.001	4.38º
Lumbar spine	25.51 ± 7.38º	27.37 ± 7.80º	1.86 ± 2.88º	−4.612	< 0.05	2.84º
Pelvic tilt	−13.31 ± 11.94º	−8.37 ± 12.09º	4.94 ± 3.13º	−11.240	< 0.001	4.60º
**Macrae & Wright test**						
Thoracic spine	71.71 ± 8.47º	71.63 ± 10.15º	−0.08 ± 8.96º	0.062	NS	3.25º
Lumbar spine	29.51 ± 8.64º	29.39 ± 8.63º	−0.12 ± 3.42 º	0.245	NS	3.33º
Pelvic tilt	46.04 ± 10.27º	46.80 ± 13.40º	−0.76 ± 6.56 º	−0.832	NS	3.94º

NS: no significant; MDC: minimal detectable change

## References

[b1-jhk-31-69] Borman NP, Trudelle-Jackson E, Smith SS (2011). Effect of stretch positions on hamstring muscle length, lumbar flexion range of motion, and lumbar curvature in healthy adults. Physiother Theory Pract.

[b2-jhk-31-69] Carregaro RL, Coury HJC (2009). Does reduced hamstring flexibility affect trunk and pelvic movement strategies during manual handling?. Int J Ind Ergon.

[b3-jhk-31-69] Congdon R, Bohannon R, Tiberio D (2005). Intrinsic and imposed hamstring length influence posterior pelvic rotation during hip flexion. Clin Biomech.

[b4-jhk-31-69] Davis DS, Ashby PE, McCale KL, McQuain JA, Wine JM (2005). The effectiveness of 3 stretching techniques on hamstring flexibility using consistent stretching parameters. J Strength Cond Res.

[b5-jhk-31-69] Delisle A, Gagnon M, Sicard C (1997). Effect of pelvic tilt on lumbar spine geometry. IEEE Trans Rehabil Eng.

[b6-jhk-31-69] DePino G, Webright WG, Arnold BL (2000). Duration of maintained hamstring flexibility after cessation of an acute static stretching protocol. J Athl Train.

[b7-jhk-31-69] de Weijer VC, Gorniak GC, Shamus E (2003). The effect of static stretch and warm-up exercise on hamstring length over the course of 24 hours. J Orthop Sports Phys Ther.

[b8-jhk-31-69] Esola MA, McClure PW, Fitzgerald GK, Siegler S (1996). Analysis of lumbar spine and hip motion during forward bending in subjects with and without a history of low back pain. Spine.

[b9-jhk-31-69] Gajdosik RL, Albert CR, Mitman JJ (1994). Influence of hamstring length on the standing position and flexion range of motion of the pelvic angle, lumbar angle, and thoracic angle. J Orthop Sports Phys Ther.

[b10-jhk-31-69] Guermazi M, Ghroubi S, Kassis M, Jaziri O, Keskes H, Kessomtini W, Hammouda IB, Elleuch MH (2006). Validity and reliability of Spinal Mouse® to assess lumbar flexion. Ann Readapt Med Phys.

[b11-jhk-31-69] Halbertsma JP, van Bolhuis AI, Göeken LN (1996). Sport stretching: effect on passive muscle stiffness of short hamstrings. Arch Phys Med Rehabil.

[b12-jhk-31-69] Hartig D, Henderson J (1999). Increasing hamstring flexibility decreases lower extremity overuse injuries in military basic trainees. Am J Sports Med.

[b13-jhk-31-69] Jones MA, Stratton G, Reilly T, Unnithan VB (2005). Biological risk indicators for recurrent non-specific low back pain in adolescents. Br J Sports Med.

[b14-jhk-31-69] Keller TS, Colloca CJ, Harrison DE, Harrison DD, Janik TJ (2005). Influence of spine morphology on intervertebral disc loads and stresses in asymptomatic adults: implications for the ideal spine. The Spine J.

[b15-jhk-31-69] Kippers V, Parker A (1987). Toe touch test: A measure of its validity. Phys Ther.

[b16-jhk-31-69] Li Y, McClure PW, Pratt N (1996). The effect of hamstring muscle stretching on standing posture and on lumbar and hip motions during forward bending. Phys Ther.

[b17-jhk-31-69] López-Miñarro PA, Alacid F, Muyor JM (2009). Comparación del morfotipo raquídeo y extensibilidad isquiosural entre piragüistas y corredores. Rev Int Med Cienc Act.

[b18-jhk-31-69] López-Miñarro PA, Alacid F, Rodriguez PL (2010). Comparison of sagittal spinal curvatures and hamstring muscle extensibility among young elite paddlers and non-athletes. Int SportMed J.

[b19-jhk-31-69] López-Miñarro PA, Muyor JM, Alacid F (2011). Influence of hamstring extensibility on sagittal spinal curvatures and pelvic tilt in high-trained young kayakers. Eur J Sports Sci.

[b20-jhk-31-69] López-Miñarro PA, Alacid F (2010). Influence of hamstring muscle extensibility on spinal curvatures in young athletes. Sci & Sports.

[b21-jhk-31-69] Macrae IF, Wright V (1969). Measurements of low back movement. Ann Rheum Dis.

[b22-jhk-31-69] Magnusson SP, Simonsen EB, Aagaard P, Kjaer M (1996). Biomechanical responses to repeated stretches in human hamstring muscle in vivo. Am J Sports Med.

[b23-jhk-31-69] Mannion AF, Knecht K, Balaban G, Dvorak J, Grob D (2004). A new skin-surface device for measuring the curvature and global and segmental ranges of motion of the spine: reliability of measurements and comparison with data reviewed from the literature. Eur Spine J.

[b24-jhk-31-69] McGill SM (2002). Low back disorders. Evidence-Based prevention and rehabilitation.

[b25-jhk-31-69] Murphy JR, Di Santo MC, Alkanani T, Behm DG (2010). Aerobic activity before and following short-duration static stretching improves range of motion and performance vs. a traditional warm-up. Appl Physiol Nutr Metab.

[b26-jhk-31-69] Muyor JM, López-Miñarro PA, Alacid F (2011). Influence of hamstring muscles extensibility on spinal curvatures and pelvic tilt in highly trained cyclists. J Hum Kinetics.

[b27-jhk-31-69] Nelson RT (2006). A comparison of the immediate effects of eccentric training vs static stretch on hamstring flexibility in high school and college athletes. N Am J Sports Phys Ther.

[b28-jhk-31-69] O’hora J, Cartwright A, Wade CD, Hough AD, Shum GL (2011). Efficacy of static stretching and proprioceptive neuromuscular facilitation stretch on hamstrings length after a single session. J Strength Cond Res.

[b29-jhk-31-69] O’Sullivan K, Murria E, Sainsbury D (2009). The effect of warm-up, static stretching and dynamic stretching on hamstring flexibility in previously injured subjects. BMC Musculoskeletal Disorders.

[b30-jhk-31-69] Peharec S, Jerković R, Bacić P, Azman J, Bobinac D (2007). Kinematic measurement of the lumbar spine and pelvis in the normal population. Coll Antropol.

[b31-jhk-31-69] Perrier ET, Pavol MJ, Hoffman MA (2011). The acute effects of a warm-up including static or dynamic stretching on countermovement jump height, reaction time, and flexibility. J Strength Cond Res.

[b32-jhk-31-69] Polga DJ, Beaubien BP, Kallemeier PM, Schellhas KP, Lew WD, Buttermann GR, Wood KB (2004). Measurement of in vivo intradiscal pressure in healthy thoracic intervertebral discs. Spine.

[b33-jhk-31-69] Puentedura EJ, Huijbregts PA, Celeste S, Edwards D, In A, Landers MR, Fernandez-de-las-Penas (2011). Immediate effects of quantified hamstring stretching: Hold-relax propioceptive neuromuscular facilitation versus static stretching. Phys Ther Sport.

[b34-jhk-31-69] Shrout PE, Fleiss J (1979). Intraclass correlations: Uses in assessing rater reliability. Psychol Bull.

[b35-jhk-31-69] Smith A, O′Sullivan P, Straker L (2008). Classification of sagittal thoraco-lumbo-pelvic alignment of the adolescent spine in standing and its relationship to low back pain. Spine.

[b36-jhk-31-69] Spernoga SG, Uhl TL, Arnold BL, Gansneder BM (2001). Duration of maintained hamstrings flexibility after a one time, modified hold-relax stretching protocol. J Athl Train.

[b37-jhk-31-69] Wilke HJ, Neef P, Hinz B, Seidel H, Claes LE (2001). Intradiscal pressure together with anthropometric data - a data set for the validation of models. Clin Biomech.

[b38-jhk-31-69] Youdas JW, Haeflinger KM, Kreun MK, Holloway AM, Kramer CM, Hollman JH (2010). The efficacy of two modified proprioceptive neuromuscular facilitation stretching techniques in subjects with reduced hamstring muscle length. Physiother Theory Pract.

